# Outcomes of hepatitis C screening programs targeted at risk groups hidden in the general population: a systematic review

**DOI:** 10.1186/1471-2458-14-66

**Published:** 2014-01-22

**Authors:** Freke R Zuure, Anouk T Urbanus, Miranda W Langendam, Charles W Helsper, Charlotte HSB van den Berg, Udi Davidovich, Maria Prins

**Affiliations:** 1 Public Health Service of Amsterdam, the Netherlands, Infectious Diseases Cluster, P.O. Box 2200, Amsterdam 1000 CE, The Netherlands; 2 Center for Infection and Immunology Amsterdam (CINIMA), Academic Medical Center (University of Amsterdam), P.O. Box 22660, Amsterdam 1100 DD, The Netherlands; 3 Dutch Cochrane Centre, Academic Medical Center, P.O. Box 22660, Amsterdam 1100 DD, The Netherlands; 4 Julius Center for Health Sciences and Primary Care, University Medical Center Utrecht, P.O. Box 85500, Utrecht 3508 GA, The Netherlands

**Keywords:** HCV, Testing, Case finding, Case detection, Reporting guidelines

## Abstract

**Background:**

Effective screening programs are urgently needed to provide undiagnosed hepatitis C virus (HCV)-infected individuals with therapy. This systematic review of characteristics and outcomes of screening programs for HCV focuses on strategies to identify HCV risk groups hidden in the general population.

**Methods:**

We conducted a comprehensive search of MEDLINE and EMBASE databases for articles published between 1991–2010, including studies that screened the general population using either a newly developed (nonintegrated) screening program or one integrated in existing health care facilities. Look-back studies, prevalence studies, and programs targeting high-risk groups in care (e.g., current drug users) were excluded.

**Results:**

After reviewing 7052 studies, we identified 67 screening programs: 24 nonintegrated; 41 programs integrated in a variety of health care facilities (e.g., general practitioner); and 2 programs with both integrated and nonintegrated strategies. Together, these programs identified approximately 25,700 HCV-infected individuals. In general, higher HCV prevalence was found in programs in countries with intermediate to high HCV prevalence, in psychiatric clinics, and in programs that used a prescreening selection based on HCV risk factors. Only 6 programs used a comparison group for evaluation purposes, and 1 program used theory about effective promotion for screening. Comparison of the programs and their effectiveness was hampered by lack of reported data on program characteristics, clinical follow-up, and type of diagnostic test.

**Conclusions:**

A prescreening selection based on risk factors can increase the efficiency of screening in low-prevalence populations, and we need programs with comparison groups to evaluate effectiveness. Also, program characteristics such as type of diagnostic test, screening uptake, and clinical outcomes should be reported systematically.

## Background

Hepatitis C virus (HCV) infection, primarily a blood-borne virus and first identified in 1989, is a major public health problem. Worldwide an estimated 130–170 million individuals are HCV-antibody positive [1], of whom approximately 75% are chronically infected and at risk for the development of cirrhosis, which can lead to liver cancer and death [2,3]. In chronically infected patients, the onset of HCV infection and the development of cirrhosis are usually asymptomatic [2,4]; many infections remain undetected or are diagnosed at a late stage. In the United States of America (USA), an estimated 43% to 72% of HCV infections are undiagnosed [5-7]. In 2001, successful combination therapy for HCV became widely accessible [8,9] and more effective therapeutic options are becoming available [10,11]. Effective screening programs are urgently needed to provide undiagnosed HCV-infected individuals with therapy and to spread information about preventive measures that each person should take, thus decreasing future morbidity and mortality.

There are several types of screening strategies such as mass population screening, selective screening, or case finding (i.e., opportunistic screening [12]). Selective screening of risk groups for HCV (see below) has been recommended [13,14]. Some of the high risk groups for HCV are relatively easy to reach and have been targeted by screening programs as part of specialized medical care (e.g., current drug users (DUs) on methadone treatment who have injected drugs in the past [15], hemophiliacs [16], and HIV-infected individuals receiving clinical care [17]). However, other risk groups are more difficult to target for screening. For example, persons at risk for HCV infection through occasional injecting drug use (IDU) in the remote past will not attend programs targeted at active injecting drug users and might not identify themselves as being at risk for HCV infection. The same holds true for individuals who received a blood transfusion before 1992. These groups can be considered as ‘hidden risk groups’ among the general population. The size of this hidden population may be substantial. A recent study estimated that of the total population of HCV-infected individuals in a high-income country, only 34% are in relatively easy to reach high-risk groups such as hemophiliac patients, HIV-infected patients, and persons with a history of IDU; 41% are first-generation migrants and 25% belong to other risk groups [18].

**Risk groups for HCV infection**[13,14]

• Individuals with a history of injecting drug use (IDU), including those who injected only a limited number of times many years ago and do not consider themselves to be drug users

• Individuals who received clotting factor concentrates produced before 1987 or a blood transfusion or an organ transplant before 1992 including hemophiliac patients (systematic screening of blood donors for HCV antibodies was introduced in 1991) [19]

• Individuals with occupational exposure to infected blood

• People living with HIV

• Chronic hemodialysis patients

• Children born to HCV-infected mothers

• Individuals exposed to HCV-infected blood through invasive procedures or blood product transfusions due to absence of precautionary measures to prevent transmission, mostly in low-resource settings. This group includes first-generation migrants.

Finding an effective strategy to identify the hidden population of undiagnosed HCV-infected individuals is challenging. An overview of HCV screening programs provides insight into strategies that have been used so far and their outcomes, and can provide insight into the best way forward. In our review, we systematically evaluate characteristics and outcomes of HCV screening programs targeted at risk groups hidden in the general population. We focused in particular on the promotion of the screening program, whether or not prescreening selection criteria were used, and the use of psychosocial theory or knowledge about determinants facilitating participation in screening programs, since health promotion programs that are based on theory are more likely to be effective than those that are not [20]. We discuss the implications of these findings for future HCV screening strategies.

## Methods

### Search strategy

We searched in the MEDLINE (PubMed) and EMBASE databases for articles published in any language before July 27, 2010. A comprehensive strategy was used to include all possible studies in which individuals were screened for HCV. Search terms included hepatitis C (Medical Subject Headings [MeSH] for PubMed and Explosion search [Exp] for Embase) or HCV or “hepatitis C” in title or abstract combined with search terms in title or abstract that reflect screening (i.e., mass screening [MeSH/Exp], screen*, “case finding*”, “case identification*”, “case detection*”, “hepatitis C testing”, “HCV testing”) or search terms in title or abstract and/or MeSH/Exp that reflect campaigns or evaluation of health programs (i.e., campaign*, health promotion, health service*”, feasibility, pilot*, “program* evaluation*”, “program* effect*”, “*health care quality”). The search was limited to articles published after 1990 since a more sensitive second-generation HCV antibody test was introduced in 1991 [21]. The complete search strategy including truncation characters is available from the authors. In addition, we screened the reference lists of the articles that were included in the selection.

### Study selection

Studies were included if they reported screening of individuals in the general population, including screening in primary care facilities that are not related to specific HCV risk groups. Screening is defined as testing for HCV antibodies. Exclusion criteria included ‘look-back’ studies, in which recipients of HCV-infected donor blood are notified and offered screening and studies conducted in specific, identifiable risk groups for HCV that are in specialized care: injecting drug users, HIV-infected individuals, incarcerated individuals, hemodialysis patients, or multitransfused patients such as hemophiliac patients. In addition, studies were excluded if 1) the study was designed to assess the prevalence in a given population, and/or 2) if the study was undertaken to investigate transmission rates and determinants or the association between HCV infection and another medical condition, and/or 3) nothing was reported about notification, referral, or medical follow-up of participants (these studies were considered prevalence studies, not aiming to identify HCV infections). The latter criterion did not apply to studies describing HCV screening at the general practitioner (GP) clinic, since notification of results in this setting is considered to take place. Articles in languages other than English, Dutch, French, German, or Spanish were excluded if there was no English abstract of if the English abstract did not yield enough data.

The first selection round was based on title and abstract (if available) only and was done by four authors (FZ, AU, CH, and CvdB). The database including the titles and abstracts obtained through the search was split in four. The reviewers independently screened two of the subdatabases each so that each title/abstract was screened in duplicate. Studies were included in the second screening round if selected by at least one reviewer. The second selection round comprised screening of the full-text articles. Two authors (FZ and AU) independently screened all articles for eligibility using the aforementioned criteria. Any discrepancies were resolved by discussion until consensus was reached, and unresolved discrepancies were arbitrated by a third reviewer (MP).

### Data extraction and validity checking

Data regarding program characteristics and program outcomes (see Parameters of screening programs) were extracted and cross-checked by two reviewers (FZ and AU). If the study reported HCV prevalence, but without specifying whether it concerned HCV-antibody or HCV-RNA prevalence, and if information about the test that was used was lacking, we assumed it to be HCV-antibody prevalence. Since the studies were performed in different periods of time, different types of tests were used over the years. Therefore the prevalences were qualified depending on the test used. Anti-HCV and HCV-RNA prevalence rates of studies were considered suboptimal 1) if data was collected prior to 1994 when sensitivity and specificity of tests were not optimal [19,22] or 2) if studies did not confirm reactive HCV antibody test results by immunoblot or PCR to eliminate false positives. Tests were considered valid if performed after 1993 and if 1) second- or higher- generation immunoblot assays from Ortho, Chiron, Novartis (RIBA), Innogenetics (LiaTek), Pasteur (DECISCAN HCV), Genelabs Diagnostics (HCV BLOT), or Mikrogen (recomBlot HCV IgG 2.0) were used to confirm HCV antibody reactive results or 2) PCR was used to confirm HCV- antibody reactive results. The validity of outcomes of studies that did not indicate which test was used, and studies that used dried blood spot (DBS), oral fluid screening, or immunoblot assays different from those indicated above, was considered undecided.

We distinguished two types of settings and presented the screening programs according to these: integrated and nonintegrated screening. Integrated screening refers to programs that are integrated within already existing health care facilities, whereas in nonintegrated screening, the program is exclusively set up for the screening. In addition, since screening strategies may differ according to the HCV prevalence in a specific country, data are presented not only by the type of setting, but also separately for low HCV prevalence (≤2%) ) and intermediate to high HCV-prevalence countries (>2%; country-specific prevalence estimates were obtained from reference [23]).

Parameters of screening programs

Program characteristics

• Country (and region, if applicable) of the study

• Estimated HCV antibody prevalence in the country

• Calendar year(s) of data collection

• Duration of enrolment/screening period

• Setting (i.e., whether HCV screening was integrated within already existing health care facilities or whether the program was exclusively set up for the screening [i.e., nonintegrated screening])

• Use of psychosocial theory or previous research findings as a basis for communicating the screening and for stimulating screening uptake

• Size of the targeted population

• Use of media activities and/or personal invitations to promote screening

• Use of screening criteria based on HCV risk factors

• Incentive or participant’s costs for screening

• Anonymous or nonanonymous participation

• Type of HCV test(s) that was used for screening

• Screening for other diseases performed

• Use of a comparison group for evaluation purposes

Program outcomes

• Response rate (i.e., proportion of the target population that was screened)

• Number of participants (i.e., number of individuals that were screened)

• Number of HCV cases identified

• Number of HCV cases already known

• HCV-antibody prevalence

• Risk profile of identified cases

• Proportion of HCV-antibody positives with detectable HCV RNA

• Number of referrals to specialist

• Start and outcomes of treatment

## Results

The search strategy identified 5,263 records from the MEDLINE database and 6,300 from the EMBASE database. After duplicates were eliminated, 7,052 of 11,563 records remained. Of those, 737 were selected as potentially relevant to the review, and full-text articles were retrieved and reviewed independently in duplicate. We excluded 677 articles; 652 articles because they did not meet the inclusion criteria (the majority because they were prevalence studies, or studies that only reported statements about HCV screening guidelines and policy, not including any screening results), and 3 Japanese articles and 1 Italian because they did not provide an English abstract. In addition, 20 articles (two Chinese [24,25], eight Japanese [26-33], one Icelandic [34], four Russian [35-38], two Turkish [39,40], one Czech [41], and two Taiwanese [42,43]) seemed relevant on the basis of the English abstracts, but were excluded as the abstracts alone did not yield enough information for review. One article was excluded because the same data were reported in two papers [44,45]. Of the 60 studies remaining, references lists were screened yielding an additional 106 potentially relevant records. The full-text articles were retrieved and screened independently in duplicate, and 7 of the 106 studies were selected for inclusion. In total, 67 studies remained in the final selection.

The 67 studies identified were done in the USA (n = 27), Europe (n = 27; mostly France and the United Kingdom [UK]), Asia (n = 4), Australia (n = 4), South America (n = 3), Egypt (n = 1) and Saudi Arabia (n = 1). We identified 24 nonintegrated and 41 integrated studies, plus two studies that used both strategies (the latter are shown in the paragraph below for the results of the nonintegrated part of their program, and in the subsequent paragraphs for the results of the integrated part of their program) [46,47]. A total of 85% (22/26) of the non-integrated programs and all of the integrated programs were from low HCV-prevalence countries.

### Nonintegrated HCV screening programs in low HCV-prevalence countries (n = 22)

#### Program characteristics

Additional file 1: Table S1 presents the 20 nonintegrated HCV screening programs and the 2 programs that combined an integrated and non-integrated screening approach that were performed in low HCV prevalence countries. In total, 12 of the 22 programs were carried out in the USA. The table is sorted by population type; seven studies were aimed at screening the general population; the other 15 studies targeted specific groups in which a higher HCV prevalence might have been expected (e.g., migrants, homeless individuals, firefighters, surgeons). Five of the 22 programs reported the use of personal screening invitations either face to face or by mail, and 12 reported the use of media activities to attract individuals for screening. Eight studies reported the possibility for individuals to participate anonymously. Only nine of 22 studies reported the costs for participants to be screened; in all of them, screening was offered free of cost, and one study offered a t-shirt as an incentive for screening [48].

With respect to screening procedures, except for two, all programs used venipuncture to collect serum. A program targeted at firefighters [49] used home specimen collection kits for serum collection. A program targeted at migrants [46] initially used oral fluid HCV antibody tests followed by a blood test for those who tested positive (no further details reported). In the majority (16/22) of the programs, participants were also screened for other infections (mostly HIV and hepatitis B virus [HBV]) or elevated liver enzymes (alanine aminotransferase [ALT], aspartate aminotransferase [AST]).

#### Program outcomes

The number of individuals screened for HCV antibodies per program ranged from 19 to 8,650. The screening uptake was reported in 13/22 studies, and varied from >20% in a screening program at a local health fair in the USA [50] to 100% in a program that used household visits to identify transfusion recipients and invite them for screening in Cuba [51]. The HCV prevalence varied from 0% to 28.3%. The latter was found in a community-based screening program in New York City targeted at migrants from the former Sovjet Union. HCV risk-profile data were available for only a subset of the HCV-infected individuals in that study and included intramuscular injections and blood transfusions. Some of the programs among a so-called ‘general population’ (see Additional file 1: Table S1, row 1–7) that found relatively high HCV prevalences (e.g., 10.5% in a walk-in clinic [52]), did not collect risk profile data of their participants, limiting the interpretability of their findings. Two of 22 studies used a prescreening risk assessment in order to limit screening to those with established HCV risk factors: one did not report the prevalence nor screening uptake [53]; the study in Cuba reported the highest screening uptake (100%) and found relatively high HCV prevalence (8.6%), but absolute numbers were small.

Four of the 22 programs screened primarily people from Asia, either through screening programs in Asia (Japan), or programs in Western countries targeting Asian migrants. In all but one of these programs, relatively high HCV prevalences were found, varying from 5.2% to 19.7%. In contrast, the programs targeting those with occupational risk for HCV (n = 7) found relatively low HCV prevalences (all <1.1%, except for 3.6% among firefighters and 5.3% among health care workers involved with liver transplantations).

We did not notice clear differences in screening uptake or HCV prevalence related to the use of personal invitations for screening, the use of media to attract individuals for screening, and whether or not individuals were screened for other infections as well. In general, a lower HCV prevalence was found in the studies (n = 8) that provided anonymous screening for HCV; however, most of these studies (6/8) targeted those with occupational risk for HCV, explaining the lower prevalence.

Only one study compared the results of their outreach screening program with data collected in the same period at a screening clinic that is visited by individuals on their own initiative [54]. A higher prevalence of HCV was found during outreach screening (4.9% versus 1.6%, respectively). However, the number of individuals that returned to obtain their test results was much lower for the outreach approach (65.8% versus 91.8%, respectively). Only one study reported the proportion of identified HCV-infected individuals that started treatment (37%) [53], but did not report how many of those reached a sustained virological response (SVR).

### Nonintegrated HCV screening programs in intermediate to high HCV-prevalence countries (n = 4)

#### Program characteristics

Table 1 presents the four nonintegrated HCV screening programs that were performed in intermediate to high HCV-prevalence countries (Taiwan [n = 2], Pakistan [n = 1] and Egypt [n = 1]). All studies targeted the general population; one targeted children less than 16 years of age. A study from Egypt reported household visits to personally invite individuals for screening [55]; the study among children reported personal invitations (method not specified) [56]. All except the study among children reported the use of media activities to attract individuals for screening. A risk-based screening selection was used in the program in Egypt, where screening was limited to those with symptoms and ALT levels ≥ 2 times the upper limit of normal. None of the studies reported the possibility for individuals to participate anonymously. Two studies reported about the costs for participants to be screened; one of them offered screening free of cost, whereas the other program [57] offered screening at 20% of the market value. None of the studies used a comparison group for evaluation purposes. With respect to screening procedures, all four studies used venipuncture for specimen collection. A study from Pakistan [57] used a rapid HCV antibody test. In most (3/4) studies, participants were also screened for other infections (mostly HBV) but not for HIV.

**Table 1:** Nonintegrated screening programs in intermediate to high HCV-prevalence countries (>2%)

**Program characteristics**									**Program outcomes**		
**First author, year of publication**	**Calendar year of data collection**	**Population**	**Country and HCV prevalence according to CDC**[23]	**Setting of screening**	**Duration of screening program**	**Other tests**	**Pre-screening selection**	**Media activities**	**Screening uptake and anti-HCV prevalence (95% CI)**	**Risk profile of identified HCV cases/Risk factors associated with HCV**	**Follow-up of HCV-infected individuals**
Meky et al. 2006 [55]	2002-2005	General population	Egypt (6.6%): Two rural villages in the Nile Delta	Community health clinic and private clinics for acute cases	29 months	HAV, HBV, HEV, CMV, Epstein-Barr	Only those with symptoms and ALT levels = > 2 times the upper limit of normal were tested	Yes	Scr. uptake: NR Prevalence: 78.7% (37/47; 95% CI:65.1-88.0*	NR	At 2 and 6 months following initial examination, follow-up testing was done to confirm or reclassify the diagnosis (i.e., viral clearance or persistent infection). No data was reported about medical follow-up of chronically infected patients.
											Outcomes: RNA rate: 70.2% (33/37) Start treatment: NR SVR: NR
Chen et al. 2007 [58]	1996-2005	General population aged ≥18 yrs	Taiwan (2.1% ^a^): throughout the country	Outreach community based screening	10 years	HBV, ALT, AST	No	Yes	Scr. uptake: NR Prevalence: 4.4% (6904/157720; 95% CI: 4.3-4.5)**	NR	Patients were requested to return to the collaborating hospitals for subsequent management (results were not reported).
											Outcomes: RNA rate: NR Start treatment: NR SVR: NR
Aslam & Aslam 2001 [57]	2000	General population	Pakistan (6.6%): Lahore and Gujranwala	City screening program	NR	None	No	Yes	Scr. uptake: Lahore: 0.01% 488/5063500 Gujranwala: 0.2% (1922/1124800) Prevalence: Lahore: 16% (78/488; 95% CI:13.0-19.5) Gujranwala: 23.8% (458/1922; 95% CI: 22.0-25.8)*	Listed risk factors: - Blood transfusion - Surgery/dental work - Multiple factors - Mostly other, non-specified risk factors	Patients were informed about the possibility of eradication of the virus, and treatment in its early stages (further data not provided) Outcomes: RNA rate: NR Start treatment: NR SVR: NR
Lu et al. 1998 [56]	1994	General population <16 yrs	Taiwan (2.1% ^a^): Paisha Township, Penghu Islets	Kindergartens and schools	1 month	HBV	No	NR	Scr. uptake: 93.6% (1164/1243) Prevalence: 0.9% (11/1164; 95% CI: 0.5-1.7) overall* 3–6 yrs: 0% 7–12 yrs: 0.8% 13–15 yrs: 1.9%	Listed risk factors: - Surgery - Intramuscular injection - Intravascul ar injection, - Intravascular infusion	All anti-HCV positive children were followed annually for 2 years with upper abdominal sonography, AST, ALT, anti-HCV and HCV RNA. No data was reported about medical follow-up of chronically infected children.
											Outcomes: RNA rate: 27.3% (3/11) Start treatment: NR SVR: NR

Note: CI = confidence interval; NR = not reported; IDU = injecting drug use; HCV = hepatitis C virus; HBV = hepatitis B virus; HAV = hepatitis A virus; HEV = hepatitis E virus; CMV = cytomegalovirus; ALT = alanine aminotransferase; AST = aspartate aminotransferase; SVR = sustained virological response.

*HCV-antibody prevalence is considered suboptimal (data were collected before 1994 when sensitivity/specificity of tests was not optimal, or reactive HCV-antibody test results were not confirmed by immunoblot).

**The reliability of the reported HCV-antibody prevalence is undecided (data were collected after 1993, but the diagnostic tests are unspecified, or other than described below (see ***), or dried blood spots or oral fluid samples were used).

***HCV-antibody prevalence is considered valid; data were collected after 1993, and reactive HCV-antibody test results were confirmed by second or higher generation immunoblot assays from Ortho, Chiron, Novartis (RIBA), Innogenetics (LiaTek), Pasteur (DECISCAN HCV), Genelabs Diagnostics (HCV BLOT), or Mikrogen (recomBlot HCV IgG 2.0).

^a^HCV prevalence based on prevalence of country neighbours.

#### Program outcomes

The number of individuals screened for HCV antibodies per program ranged from 47 to 157,720. The screening uptake was reported in two studies; it was very low (<1%) in a city screening program in Pakistan [57], and very high (93.6%) in a screening program in kindergartens and schools in Taiwan [56]. Although the screening uptake in the latter was high, the prevalence was low (0.9%). The HCV prevalences in the other programs varied from 4.4% in a community-based screening program in Taiwan [58] up to 78.8% in a program in Egypt that limited screening to those with symptoms and increased ALT levels [55]. None of the studies reported the proportion of HCV-infected patients that started treatment and/or reached SVR.

### Integrated HCV screening programs

We identified 41 HCV screening programs in the following clinics that offer care not related to liver disease: sexually transmittable diseases (STD) clinics (n = 11); GP clinics (n = 10, including two programs that also used a nonintegrated approach); Veterans Affairs (VA) clinics (n = 5); antenatal/obstetric/fertility clinics (n = 5); clinics for psychiatric patients (n = 3); and other clinics or services (n = 7). Tables 2, 3, 4, 5, 6 and 7 present the programs separately for each type of setting. All programs were carried out in low HCV-prevalence countries.

**Table 2:** Integrated screening programs at clinics for sexually transmitted diseases (STD)

**Program characteristics**									**Program outcomes**		
**First author, year of publication**	**Calendar year of data collection**	**Population**	**Country and HCV prevalence according to CDC**[23]	**Setting of screening**	**Duration of screening program**	**Other tests**	**Prescreening selection**	**Media activities**	**Screening uptake and anti-HCV prevalence (95% CI)**	**Risk profile of identified HCV cases/Risk factors associated with HCV**	**Follow-up of HCV-infected individuals**
D'Souza et al. 2003 [59]	2001	STD clinic clients	USA (1.9%): Houston	STD clinic	9 months	STD	Yes, risk assessment questionnaire. screening offered to high-risk groups ^a^	NR	Scr. uptake: 95.8% (822/859) Prevalence: 15.3% (126/822; 95% CI:12.7-17.7)*	Multivariable regr. analysis: - History of IDU - Age ≥25 yrs - Heroin use - Non-transfusion/ transplantation blood exposure - Shared straw to snort drugs	Patients were referred to appropriate settings for follow-up (no results were reported). Outcomes: RNA rate: NR Start treatment: NR SVR: NR
Scott et al. 2010 [60]	2007	STD clinic clients	UK (1.1%): London, Chelsea and Westminister hospital	STD clinic in hospital	6 months	STD	Yes, MSM	NR	Scr. uptake: 68.6% (2309/3365) Prevalence: 0.6% (15/2309; 95% CI:0.4-1.1%)**	Listed risk factors: - HIV+ - History of IDU - Unprotected anal intercourse	HCV RNA was tested in 13/15 HCV antibody positive persons. Outcomes: RNA rate: 69.2% (9/13) Start treatment: NR SVR: NR
Weisbord et al. 2003 [61]	2001	STD clinic clients	USA (1.9%): Miami	STD clinic	3 months	HAV, HBV	No	NR	Scr. uptake: 50.3% (687/1365) Prevalence: 4.7% (32/687; 95% CI: 3.3-6.5)***	Multivariable regr. analysis: - History of IDU - Sex with HCV + person - Spent ≥ 1 day in prison - Older age	Patients received their test results within two weeks (no further results reported). Outcomes: RNA rate: NR Start treatment: NR SVR: NR
Gunn et al. 2003 [62]	1999-2000	STD clinic clients	USA (1.9%): San Diego	STD clinic	8 months	STD	No	No	Scr. uptake: NR Prevalence: 4.9% (165/3367; 95% CI: 4.2-5.7)***	Multivariable regr. analysis: - History of IDU - Age ≥30 yrs - Ever in jail - Blood transfusion before 1992 - History of bacterial STD - IDU sex partner	Post-test counseling was offered. A list of medical care resources was provided. In total, 136/165 were interviewed of whom 44% had no medical insurance, but 87% planned to have a medical evaluation.
											Outcomes: RNA rate: NR Start treatment: NR SVR: NR
Mapagu et al. 2008 [63]	2000-2002	STD clinic clients	Australia (2%): Canberra	STD clinic	3 years	STD, BBV	No	NR	Scr. uptake: 46.0% (3113/6774) Prevalence: 3.1% (95/3113; 95% CI: 2.5-3.7%)**	Listed risk factors: - History of IDU - Tatoos - Body piercings - Blood transfusion - IDU partner - Needle stick - Mother HCV+ - Medical treatment overseas in childhood - Prison	Of the 95 HCV antibody positive persons, 47 were tested for HCV RNA. Outcomes: RNA rate: 61.7% (29/47) Start treatment: NR SVR: NR
Zimmerman et al. 2007 [64]	2001-2005	STD clinic clients	USA (1.9%): Illinois excluding Chicago	STD clinics	5 years	HBV (only 2001), STD	Yes: 2001: IDU and snorting drugs were criteria. From 2002: only IDU	NR	Scr. uptake: NR	Listed risk factors: - Mainly IDU	Because of inadequate resources, the referrals and follow-up were not monitored.
									Prevalence: 21.2% (646/3042; 95% CI: 19.8-22.7)***		
											Outcomes: RNA rate: NR Start treatment: NR SVR: NR
Subiadur et al. 2007 [65]	2000-2005	STD clinic clients	USA (1.9%): Denver	STD clinics	6 years	STD, HIV	Yes: IDU, HCV-infected sex partner, blood transfusion before 1992	NR	Scr. uptake: NR	NR	Patients were referred to a specialist but a sub-study of 65/467 clients showed that <20% followed through with recommended services.
									Prevalence: 28.0%. (467/1666; 95% CI: 25.9-30.2)**		
											Outcomes: RNA rate: NR Start treatment: NR SVR: NR
Heseltine & McFarlane 2007 [66]	2000-2005	Not specified; clients of the various settings	USA (1.9%): Texas	Several HIV/STD service providers: HIV counseling and testing sites; drug treatment facilities, corrections facilities, field visit/outreach sites (e.g., bars, adult bookstores, homeless shelters), STD clinics, family planning clinic, primary health care facility	6 years	STD, HIV	Yes: IDU, sharing equipment used to snort drugs; having received a tattoo or piercing under unsanitary conditions; having 50 or more lifetime sex partners; exchanging sex for money; having sex with an HCV-positive person; people with some medical exposures and occupations	No	Scr. uptake: NR Prevalence: 23.2% (8964/38717; 95% CI: 22.7-23.6)***	Listed risk factors: - History of IDU (main risk factor) - Risky tattoo/piercing - Risky sex - Blood or medical exposure - Sharing snorting equipment - Occupational exposure	Post-test counseling and referral to public and private providers in the local community was offered. 50.3% of 776 substance abuse referrals, 22.4% of 4410 medical evaluation referrals, and 17.4% of 2299 vaccination referrals were confirmed. Outcomes: RNA rate: NR Start treatment: NR SVR: NR
Gunn et al. 2001 [67]	1998	STD clinic clients	USA (1.9%): San Diego	STD clinic	6 weeks	HBV	No	No	Scr. uptake: 82.4% (618/750) Prevalence: 3.4% (21/618; 95% CI: 2.2-5.1)**	Listed risk factors: - History of IDU - Among non-IDU: age ≥30 yrs	HCV-positive persons were given information about the prevention of HCV transmission and a list of facilities where they might obtain a medical evaluation. Appr. 8 months after the project, 14/21 clients were contacted of whom 6 had seen a physician for medical evaluation.
											Outcomes: RNA rate: NR Start treatment: NR SVR: NR
Ellks 2010 [68]	2008	Visitors of sexual and reproductive health service	UK (1.1%): Crewe	STD and reproductive Health Service (integrated service)	1 year	HIV, syphilis, HBV	No	No	Scr. uptake: NR Prevalence: 0.1% (8/5468; 95% CI: 0.1-0.3)**	Listed risk factors: - History of IDU - MSM who snorted drugs	There were follow-up appointments for those who tested positive. Outcomes: RNA rate: NR Start treatment: NR SVR: NR
Tweed et al. 2010 [69]	2002-2007	Visitors of STD and contraception and sexual health clinics, and specialist HIV services	UK (1.1%): Throughout the country	STD clinics, contraception and sexual health clinics, specialist HIV services	6 years	Likely STD/HIV	No	NR	Scr. uptake: estimated at 14.0% Prevalence: 3.2% (285 8/90424; 95% CI: 3.04-3.27)**	Multivariable regr. analysis: - Male sex - Age 35+ - History of IDU	Those who tested positive were followed-up with clinicians (no data reported).
											Of the antiHCV positive individuals, 60.1% (1719/2858) were tested for HCV RNA.
											Outcomes: RNA rate: 69.2% (1191/1719) Start treatment: NR SVR: NR

Note: CI = confidence interval; NR = not reported; STD = sexually transmitted disease; BBV = blood-borne virus; IDU = injecting drug use; HCV = hepatitis C virus; HBV = hepatitis B virus; HAV = hepatitis A virus; HIV = human immunodeficiency virus; MSM = men who have sex with men; SVR = sustained virological response.

*HCV-antibody prevalence is considered suboptimal (data were collected before 1994 when sensitivity/specificity of tests was not optimal, or reactive HCV-antibody test results were not confirmed by immunoblot).

**The reliability of the reported HCV-antibody prevalence is undecided (data were collected after 1993, but the diagnostic tests are unspecified, or other than described below, or dried blood spots or oral fluid samples were used).

***HCV-antibody prevalence is considered valid; data were collected after 1993, and reactive HCV-antibody test results were confirmed by second or higher generation immunoblot assays from Ortho, Chiron, Novartis (RIBA), Innogenetics (LiaTek), Pasteur (DECISCAN HCV), Genelabs Diagnostics (HCV BLOT), or Mikrogen (recomBlot HCV IgG 2.0).

^a^History of IDU, body piercing/tattooing in unsanitary conditions, transfusion recipients before 1987, needlestick injury, hemodialysis patients, those born to mothers with documented HCV infection, individuals who reported ever having been told that they were infected with HCV yet lacked supporting documentation.

**Table 3:** Integrated screening programs at general practitioner (GP) clinics

**Program characteristics**									**Program outcomes**		
**First author, year of publication**	**Calendar year of data collection**	**Population**	**Country and HCV prevalence according to CDC**[23]	**Setting of screening**	**Duration of screening program**	**Other tests**	**Pre-screening selection**	**Media activities**	**Screening uptake and anti-HCV prevalence (95% CI)**	**Risk profile of identified HCV cases/Risk factors associated with HCV**	**Follow-up of HCV-infected individuals**
Monnet et al. 2000, 2009 [70]	1997-1998	Patient of GP clinics, health centres, occupational physicians, prison health service, public laboratories	France (1.1%): Le Doubs	GP clinics, health centre, occupational physicians, prison health service, public laboratories	1 year	None	Yes, no previous positive HCV serology, and at least having one risk factor: transfusion before 1991, (ex-)IDU, (ex-)snorting cocaine, tattoo, HCV diagnosis in living environment	Yes	Scr. uptake: 82.5% (782/948) Prevalence: 4.0% (31/782; 95% CI: 2.8-5.6)*	Multivariable regr. analysis: - Age 30+ - Drug use	70.9% (22/31) attended the hepatologist for HCV RNA testing. Of those who tested HCV RNA positive, 10 had elevated ALAT of which 8 had a biopsy. Based on the results, 5 were indicated for treatment (no results reported).
											Outcomes: RNA rate: 86.4% (19/22) Start treatment: NR SVR: NR
Anderson et al. 2009 [71]	2003-2004	GP patients aged 30–54 yrs	Scotland (1.1%): socio-economically deprived area of Glasgow	GP clinics (intervention clinic and comparison clinic)	6 months	None	No; in intervention practice all individuals aged 30–54 yrs who attended non-urgent appointments were offered HCV screening. In comparison practice, no intervention was carried out.	Eligible patients in intervention practice received information leaflet	Scr. uptake: Intervention clinic: 27.8% (117/421) Comparison clinic: not applicable Prevalence: Intervention clinic: 12.8% (15/117; 95% CI: 7.9-20.0) Comparison clinic: No individuals were tested during study period.**	Multivariable regr. analysis - History of IDU	HCV RNA positive patients (n = 11) were referred to a specialist and all attended ≥1 appointment. Four years later, 8 were lost to follow-up, 2 started treatment, and 1 achieved an SVR. Outcomes: RNA rate: 73.3% (11/15) Start treatment: 18.2% (2/11) SVR: 50.0% (1/2)
Pauti et al. 2008 [44]	2007	People with poor access to health care, mostly migrants	France (1.1%): Saint-Denis and Paris areas	Health care and advice centers of Médecins du Monde^b^	4 years, but data from 1 year are reported	HIV, HBV	No	No	Scr. uptake: NR Prevalence: 5.9% (70/1196; 95% CI: 4.7-7.3)**	Listed risk factors: - North and Middle Africa - Sub-Saharan Africa - Eastern Europe	The objective of the project was to offer full access to treatment, but actual results are not reported
											Outcomes: RNA rate: NR Start treatment: NR SVR: NR
Uddin et al. 2010 [46]^a^	NR	GP patients	UK (1.1%): East London, West London, Walsall, Sandwell, Bradford	GP clinic (and at community centers, see Additional file 1: Table S1)	NR	HBV	Yes, immigrants from the Indian sub-continent (India, Bangladesh or Pakistan)	No	Scr. uptake: NR	Not applicable	Not applicable
									Prevalence: 0% (0/171; 95% CI: 0–2.19 )**		
Kallman et al. 2010 [47]^a^	NR	GP patients	USA (1.9%): Northern Virginia	GP clinic (and general health screening at Asian health fairs, see Additional file 1: Table S1)	NR	HBV	Yes, Vietnamese	NR	Scr. uptake: NR Prevalence: 1.2% (3/245; 95% CI: 0.4-3.5) at GP clinic**	Univariable regr. analysis: - Elevated AST	Patients were seen by their primary care givers for further management, or referred for further follow-up and treatment (no results reported).
											Outcomes: RNA rate: NR Start treatment: NR SVR: NR
Ouzan, D, 2003 [72]	2000	GP patients aged over 18 years	France (1.1%): Alpes-Maritimes district	GP clinic	1 month	None	Yes: unknown HCV serology status, and at least one risk factor: blood transfusion before 1991, injecting or nasal DU, incarceration. Previously diagnosed HCV positives that visited the GP in the screening period were also followed-up.	No	Scr. uptake: 66.0% (233/353) Prevalence:3.9% (9/233; 95% CI: 2.0-7.02) 9 were newly identified through screening; 229 were found with a previous diagnosis**	NR for newly identified	Patients were followed-up by their GP, referred to a specialist, or a hospital unit.
											Follow-up data are reported for newly and previously diagnosed patients together and available for 159/238. HCV RNA test results were known for n = 106, and 82 were HCV RNA positive. A liver biopsy was performed in 62, of which 31 received treatment. Treatment was effective for 10, fairly effective for 12, and ineffective for 7.
											Outcomes: RNA rate: 77.4% (82/106) Start treatment: 37.8% (31/82) SVR: NR
Josset et al. 2004 [73]	1997	GP patients aged 18–70 yrs	France (1.1%): Haute-Normandie	GP clinic	10 days	None	Yes, no previous HCV serology performed, and at least one of the traditional risk factors present ^c^	No	Scr. uptake: 72.7% (3550/4883) Prevalence: 1.4% (49/3550; 95% CI: 1.0-1.8%)**	An evaluation of screening strategies based on risk factor data showed that screening those with a history of blood transfusion or drug use appeared to be the most efficient approach.	NR Outcomes: RNA rate: NR Start treatment: NR SVR: NR
Pradat et al. 2001 [74]	1997	GP patients aged 18–69 yrs	France (1.1%): Lyon area	GP clinics	6 months; each GP clinic offered HCV screening for 5 days	None	No	NR	Scr. uptake: 59.0% 6876/11646 Prevalence: 0.4% (30/6876; 95% CI: 0.3-0.6)***	Listed risk factors: - History of IDU - Transfusion <1990 - Other risk factors	NR Outcomes: RNA rate: NR Start treatment: NR SVR: NR
Altman et al. 1999 [75]	1997	GP patients	France (1.1%): Val-de-Marne and Hauts-de-Seine	GP clinics	2 weeks	None	Yes, no previous HCV serology performed, and a history of IDU or transfusion before 1991	No	Scr. uptake:	NR	NR
									Transfusion group: 76.9% (226/294) History of IDU group: 50.0% (13/26)		Outcomes: RNA rate: NR Start treatment: NR SVR: NR
									Prevalence: Transfusion group: 3.1% (7/226, 95% CI: 1.5-6.3) History of IDU group: 30.8% (4/13; 95% CI: 12.7-57.6)*		
Helsper et al. 2010 [76]	2007-2008	GP patients	Netherlands (1.1%): Amersfoort and Apeldoorn	GP clinics in intervention region with primary care practice support, and GP clinics in control region without practice support	4 months	None	Yes, individual risk estimation by GP	Yes	Scr. uptake: NR	Data not available	NR
									Prevalence: Intervention: 1.7% (3/172; 95% CI: 0.6-5.0) Control: 0.8% (1/118; 95% CI: 0.04-4.6)**		Outcomes: RNA rate: NR Start treatment: NR SVR: NR
	
Sahajian et al. 2004 [77]	2000-2001	GP, private practitioners, and specialist patients	France (1.1%): Lyon area	GP clinics Intervention: A campaign including training aimed at GPs was designed to improve screening practices. The campaign also reached the public. Comparison: Data were compared to the 12-months period preceding the campaign.	12 months	None	Yes, history of IDU, blood products before 1991, or elevated serum transaminase levels	Yes	Scr. uptake: NR	NR	NR
									Prevalence: Intervention: 1.73% (276/15952; 95% CI: 1.53-1.94) Comparison: 1.67% (231/13799; 95% CI:1.47-1.90)**		Outcomes: RNA rate: NR Start treatment: NR SVR: NR
	
Roudot-Thoraval et al. 2000 [78]	1997-1998	GP patients aged 6–85 years	France (1.1%): Le Doubs and n^o^ 6 d’Ile-de-France	GP clinics Intervention 1: GPs asked their patients for risk factors for HCV and offered screening to those at risk. Intervention 2: Posters and leaflets in GPs waiting rooms, motivating those with a risk to discuss testing with their GP.	15 months	None	Yes, several risk factors (not all are listed), among which a history of IDU, transfusion, tattoo, HCV in social environment	Yes (intervention 2)	Scr. uptake: NR Prevalence: Intervention 1: 5.7% (15/261; 95% CI: 3.51-9.26) Intervention 2: 4.4% (10/228; 95% CI: 2.40-7.88)**	Listed risk factors: - History of IDU - Transfusion before 1991 - Elevated ALT or symptoms - Tattoo - Other	NR Outcomes: RNA rate: NR Start treatment: NR SVR:

Note: CI = confidence interval; NR = not reported; IDU = injecting drug use; HCV = hepatitis C virus; HBV = hepatitis B virus; HIV = human immunodeficiency virus; ALT = alanine aminotransferase; AST = aspartate aminotransferase; SVR = sustained virological response.

*HCV-antibody prevalence is considered suboptimal (data were collected before 1994 when sensitivity/specificity of tests was not optimal, or reactive HCV-antibody test results were not confirmed by immunoblot).

**The reliability of the reported HCV-antibody prevalence is undecided (data were collected after 1993, but the diagnostic tests are unspecified, or other than described below, or dried blood spots or oral fluid samples were used).

***HCV-antibody prevalence is considered valid; data were collected after 1993, and reactive HCV-antibody test results were confirmed by second or higher generation immunoblot assays from Ortho, Chiron, Novartis (RIBA), Innogenetics (LiaTek), Pasteur (DECISCAN HCV), Genelabs Diagnostics (HCV BLOT), or Mikrogen (recomBlot HCV IgG 2.0).

^a^These programs combined a nonintegrated screening approach with integrated screening at the GP clinic (see Additional file 1: Table S1). Here only results of the integrated screening are presented.

^b^Médecins du Monde (‘Doctors of the World’) is an international humanitarian organization providing medical care to vulnerable populations.

^c^Transfusion before 1991, history of drug use, history of gastroscopy, contact with HCV infected person (spouse or other family member, occupational exposure, active or former imprisonment, history of invasive procedures (catheterism, fluid aspiration/cytology, biopsy), history of colonoscopy, history of surgery.

**Table 4:** Integrated screening programs at VA clinics

**Program characteristics**									**Program outcomes**		
**First author, year of publication**	**Calendar year of data collection**	**Population**	**Country and HCV prevalence according to CDC**[23]	**Setting of screening**	**Duration of screening program**	**Other tests**	**Prescreening selection**	**Media activities**	**Screening uptake and anti-HCV prevalence (95% CI)**	**Risk profile of identified HCV cases/Risk factors associated with HCV**	**Follow-up of HCV-infected individuals**
Groom et al. 2008 [79]	2000-2001	Veterans	USA (1.9%): Minneapolis	Veteran Affairs clinic	2 years	None	Yes, only those with a risk factor were screened (risk factors not specified)	NR	Scr. uptake: NR	NR	In total, 520/681 were HCV RNA positive of which 430 referred to a specialist, of which 88.8% (382/430) attended an appointment. Of those, 32.5% (124/382) received treatment which was successful in 37.0% (46/124) (SVR).
									Prevalence: 5.5% (681/12485; 95% CI: 5.1-5.9)*		
											Outcomes: RNA rate: 76.4% (520/681) Start treatment: 32.5% (124/382) SVR: 37.1% (46/124)
Mallette et al. 2008 [80]	1998-2004	Veterans	USA (1.9%): Providence	GP patients presenting to VA clinics	5 years and 8 months	None	Yes, only those with a risk factor were screened^a^	NR	Scr. uptake: 66.7% (5646/8471) Prevalence: 7.3% (412/5646; 95% CI: 6.6-8.0); without already known positives: 260/5646 = 4.6% (95% CI = 4.1-5.2%)***	Listed risk factors: - History of IDU - Blood transfusion before 1992 - Intranasal cocaine use - Multiple sex partners - Tattoos	Of the newly diagnosed, 46.9% (122/260) had chronic HCV, of which 46.7% (57/122) were treatment eligible. Of those, 31.6% (18/57) received treatment and 33.3% (6/18) reached an SVR. Outcomes: RNA rate: 46.9% (122/260) Start treatment: 14.8% (18/122) SVR: 33.3% (6/18)
Cheung et al. 2006 [81]	2000-2001	Veterans	USA (1.9%): Palo Alto	VA clinic	12 months	None	Yes, if not previously tested, and if one or more risk factors were reported^b^	NR	Scr. uptake: NR	NR	In total, 362/536 patients were evaluated of which 84.8% (307/362) had chronic HCV. Of those, 18.6% (57/307) were treatment eligible of whom 24.6% (14/57) completed treatment with long-term follow-up, and 35.7% (5/14) achieved SVR.
									Prevalence: 5.0% (536/10751; 95% CI: 4.6-5.4)***		
											Outcomes: RNA rate: 84.8% (307/362) Start treatment: NR SVR: 35.7% (5/14)
Rifai et al. 2006 [82]	2000-2001	Veterans	USA (1.9%): Virginia	Rural VA clinic	22 months	None	Yes, only non-IDU substance using veterans who were admitted to a substance-use residential and rehabilitation treatment program were tested	NR	Scr. uptake: 99.4% (338/340) Prevalence: 23.1% CHCV (78/338; 95% CI: 18.9-27.9) (incl. 2 who knew already)****	Univariate regr. analysis: - Cocaine snorting - History of IDU	In total, 48.7% (38/78) of the patients remained abstinent for 6 months and 30 were indicated for treatment and received treatment. In 46.7% (14/30) treatment was successful (SVR).
											Outcomes: RNA rate: n/a Start treatment: 81.1% (30/37) SVR: 46.7% (14/30)
Zuniga et al. 2006 (abstract) [83]	2001-2003	Veterans	USA (1.9%): Suffolk County, Long Island	Primary-care outpatient departments of the Northport VA clinic (suburban VA hospital)	27 months	None	Yes, only those with a risk factor were screened^c^	No	Scr. uptake: 41.9% (2263/5400) Prevalence: 4.6% CHCV (103/2263; 95% CI: 3.8-5.5)****	Multivariable regr. analysis - Age 40–54 yrs - Black race - History of IDU - Service during Vietnam era - Blood transfusion prior to 1992 - Tattoo or repeated body piercing - History of abnormal LFTs	NR Outcomes: RNA rate: n/a Start treatment: NR SVR: NR

Note: CI = confidence interval; NR = not reported; VA = veterans affairs; IDU = injecting drug use; HCV = hepatitis C virus; CHCV = chronic hepatitis C virus; HIV = human immunodeficiency virus; LFT = liver function test; SVR = sustained virological response; PCR = polymerase chain reaction.

*HCV-antibody prevalence is considered suboptimal (data were collected before 1994 when sensitivity/specificity of tests was not optimal, or reactive HCV-antibody test results were not confirmed by immunoblot).

***HCV-antibody prevalence is considered valid; data were collected after 1993, and reactive HCV-antibody test results were confirmed by second or higher generation immunoblot assays from Ortho, Chiron, Novartis (RIBA), Innogenetics (LiaTek), Pasteur (DECISCAN HCV), Genelabs Diagnostics (HCV BLOT), or Mikrogen (recomBlot HCV IgG 2.0).

****HCV-antibody prevalence is considered valid, but reflecting chronic HCV infection (data were collected after 1993, and reactive HCV antibody test results were confirmed by PCR).

^a^Vietnam-era veteran, transfusion of blood of blood products before 1992, history of IDU, history of snorting cocaine, history of 5 or more drinks a day for 10 or more years in your lifetime, history of multiple (10 or more) sexual partners in your lifetime, a man who has sex with men, history of exposure to blood on skin or mucous membranes, required chronic hemodialysis, have a tattoo or body piercing, have had a positive test for HIV or hepatitis B, have been told that you have unexplained liver disease.

^b^Blood transfusion prior to 1992, IV drug use (even once), snorting of cocaine, blood exposure, sexual promiscuity (>10 lifetime sex partners), renal dialysis, tattoo or body piercing, excessive alcohol use.

^c^Vietnam-era veteran, transfusion of blood products prior to 1992, history of IDU, blood exposure in or through skin or mucous membranes, multiple sexual partners (past or present), hemodialysis, tattoo or repeated body piercing, intranasal cocaine use (past or present), unexplained liver disease, having been told that he/she has abnormal liver function tests, intemperate alcohol use (more than seven alcoholic beverages per week).

**Table 5:** Integrated screening programs in antenatal/obstetric/fertility clinics

**Program characteristics**									**Program outcomes**		
**First author, year of publication**	**Calendar year of data collection**	**Population**	**Country and HCV prevalence according to CDC**[23]	**Setting of screening**	**Duration of screening program**	**Other tests**	**Prescreening selection**	**Media activities**	**Screening uptake and anti-HCV prevalence (95% CI)**	**Risk profile of identified HCV cases/Risk factors associated with HCV**	**Follow-up of HCV-infected individuals**
Alexanian et al. 2009 [84]	NR	Pregnant women	UK (1.1%): London	Antenatal clinic	8 years, Hospital records search	HBV, HIV	No	NR	Scr. uptake: NR	NR	In total, 73.0% (84/115) of patients had chronic HCV, of whom 55.9% (47/84) were lost to follow-up, 10.7% (9/84) deferred treatment, 4.8% (4/84) were on treatment, and 17.9% (15/84) completed treatment. Of these 15, 12 achieved SRV, 1 relapsed, and two failed to respond.
									Prevalence: 0.4 (115/31081; 95% CI: 0.3-0.4)*		
											Outcomes: RNA rate: 73.0% (84/115) Start treatment: 67.9% (19/28) SVR: 80.0% (12/15)
Abusheikha et al. 1999 [85]	1996-1998	Couples receiving fertility treatment	UK (1.1%): Cambridge	Bourn Hall clinic, infertility hospital	3 years	HIV, HBV	No	NR	Scr. uptake: NR Prevalence: 0.5% (9/1658; 95% CI: 0.3-1.0)**	NR	All patients were counseled by senior medical staff. Outcomes: RNA rate: NR Start treatment: NR SVR: NR
Leikin et al. 1994 [86]	1991-1992	Pregnant women who are at risk for perinatal complications	USA (1.9%): Valhalla	Hospital (obstetric)	19,5 months	ALT	No	NR	Scr. uptake: NR Prevalence: 4.6% (78/1700; 95% CI: 3.7-5.7)*	Multivariable regr. analysis: - History of IDU - Other drug use - Age > 30 - Incarceration - Blood transfusion	In total, 96.2% (75/78) of the patients returned for follow-up. No further details reported. Outcomes: RNA rate: NR Start treatment: NR SVR: NR
Ward et al. 2000 [87]	1997-1999	Pregnant women	UK (1.1%): London	Antenatal clinic	18 months	HBV	No	Yes	Scr. uptake: 98.0% (4727/4825) Prevalence: 0.8% (38/4729; 95% CI: 0.6-1.1)***	Univariate regr. analysis: - History of IDU - HCV-infected partner - Tattoo - Partner IDU	In total, 71.1% (27/38) had chronic HCV, and 85.2% (23/27) were offered follow-up appointments so far. Of those, 82.6% (19/23) attended for further investigations. Outcomes: RNA rate: 71.1% (27/38) Start treatment: NR SVR: NR
Costa et al. 2009 [88]	2004-2005	Pregnant women	Brazil (1%): Central Brazil, Goiania	Antenatal clinics	NR	HIV and seven other infectious diseases (not specified)	No	NR	Scr. uptake: 99.9% (28561/28576) Prevalence: 0.2% CHCV (43/28561; 95% CI: 0.1-0.2%)****	Multivariable regr. analysis: - Older age - >3 pregnancies(no data on risk factors collected)	Patients were referred for free of cost medical counseling (no results reported). Outcomes: RNA rate: n/a Start treatment: NR SVR: NR

Note: CI = confidence interval; NR = not reported; IDU = injecting drug use; HCV = hepatitis C virus; CHCV = chronic hepatitis C virus; HIV = human immunodeficiency virus; HBV = hepatitis B virus; ALT = alanine aminotransferase; SVR = sustained virological response; PCR = polymerase chain reaction.

*HCV-antibody prevalence is considered suboptimal (data were collected before 1994 when sensitivity/specificity of tests was not optimal, or reactive HCV-antibody test results were not confirmed by immunoblot).

**The reliability of the reported HCV-antibody prevalence is undecided (data were collected after 1993, but the diagnostic tests are unspecified, or other than described below, or dried blood spots or oral fluid samples were used).

***HCV-antibody prevalence is considered valid; data were collected after 1993, and reactive HCV-antibody test results were confirmed by second or higher generation immunoblot assays from Ortho, Chiron, Novartis (RIBA), Innogenetics (LiaTek), Pasteur (DECISCAN HCV), Genelabs Diagnostics (HCV BLOT), or Mikrogen (recomBlot HCV IgG 2.0).

****HCV-antibody prevalence is considered valid, but reflecting chronic HCV infection (data were collected after 1993, and reactive HCV antibody test results were confirmed by PCR).

**Table 6:** Integrated screening programs in psychiatric clinics

**Program characteristics**									**Program outcomes**		
**First author, year of publication**	**Calendar year of data collection**	**Population**	**Country and HCV prevalence according to CDC**[23]	**Setting of screening**	**Duration of screening program**	**Other tests**	**Prescreening selection**	**Media activities**	**Screening uptake and anti-HCV prevalence (95% CI)**	**Risk profile of identified HCV cases/Risk factors associated with HCV**	**Follow-up of HCV-infected individuals**
Freudenreich, O, 2007 [89]	2003-2004	Psychiatric patients (most schizophrenia)	USA (1.9%): Boston	Clozapine outpatient clinic (psychiatric patients)	4 months	None	No	NR	Scr. uptake: 100% (98/98)	Most common risk factors: - Polysubstance abuse	All patients were referred to a specialist; after two years, none had started treatment. One patient became unstable psychologically after the discovery of his infection.
									Prevalence: 8.2% (8/98; 95% CI: 4.2-15.3) (incl the one known before)*		
											Outcomes: RNA rate: 50.0% (4/8) Start treatment: 0% (0/8) SVR: -
Gunewardene, R, 2010 [90]	NR	Psychiatric patients	Australia (2%): in a capital city	Acute psychiatric inpatient unit within an Area Health service in Australia. Comparison of two strategies.	6 months	None	Unit A: No; Unit B: Yes (IDU, exposure to contaminated blood products)	NR	Scr. uptake: Unit A: 79.8% (95/119) Unit B: 90.0% (36/40) Prevalence: Unit A: 3.2% (3/95; 95% CI: 1.1-8.9);	Most common risk factors: - History of IDU	All patients were offered post-test counseling and were referred to a specialist (no results reported). Outcomes: RNA rate: NR Start treatment: NR SVR: NR
Lacey, C, 2007 [91]	2002-2003	Psychiatric patients	Australia (2%): Melbourne, Victoria	Inner city public hospital (psychiatric)	6 months	None	Yes; Patients admitted with psychotic or affective disorders, >18 yrs, inpatient stay >2 days, and did not have known HCV infection	Yes	Scr. uptake: 20.5% (71/346) Prevalence: 19.7% (14/71; 95% CI: 12.1-30.4)*	Most common risk factors: - History of IDU - Sharing injection equipment	All positive patients received post-test counseling and were referred to a specialist (no results reported). Outcomes: RNA rate: NR Start treatment: NR SVR: NR

Note: CI = confidence interval; NR = not reported; IDU = injecting drug use; HCV = hepatitis C virus; SVR = sustained virological response.

*HCV-antibody prevalence is considered suboptimal (data were collected before 1994 when sensitivity/specificity of tests was not optimal, or reactive HCV-antibody test results were not confirmed by immunoblot).

**The reliability of the reported HCV-antibody prevalence is undecided (data were collected after 1993, but the diagnostic tests are unspecified, or other than described below (see ***), or dried blood spots or oral fluid samples were used).

***HCV-antibody prevalence is considered valid; data were collected after 1993, and reactive HCV-antibody test results were confirmed by second or higher generation immunoblot assays from Ortho, Chiron, Novartis (RIBA), Innogenetics (LiaTek), Pasteur (DECISCAN HCV), Genelabs Diagnostics (HCV BLOT), or Mikrogen (recomBlot HCV IgG 2.0).

**Table 7:** Integrated screening programs integrated in other clinics or services

**Program characteristics**									**Program outcomes**		
**First author, year of publication**	**Calendar year of data collection**	**Population**	**Country and HCV prevalence according to CDC**[23]	**Setting of screening**	**Duration of screening program**	**Other tests**	**Pre-screening selection**	**Media activities**	**Screening uptake and anti-HCV prevalence (95% CI)**	**Risk profile of identified HCV cases/Risk factors associated with HCV**	**Follow-up of HCV-infected individuals**
Capron, D, 1999 [92]	1996	Patients > 14 y admitted to an emergency unit	France (1.1%):Picardy	Emergency health unit hospital	At least one week per unit (7 units) over a period of 2 months	None	Yes, only those with a reported risk factor were tested	NR	Scr. uptake: NR Prevalence: 2.4% (11/451; 95% CI: 1.4-4.3)*	Most common risk factors: - Blood transfusion - History of drug addiction - Surgery - Endoscopy	All patients were referred for medical follow-up. In total, 36.4% (4/11) attended, of which 50.0% (2/4) had chronic HCV. Liver biopsy showed minimal activity, and treatment was not indicated Outcomes:RNA rate: 50.0% (2/4) Start treatment: 0% (0/2) SVR: -
Alswaidi, FM, 2010 [93]	2008	Individuals from the general population that wish to get married	Saudi Arabia (0.9%): throughout the country	Mandatory premarital national screening program	4 months	HBV, HIV	No	NR	Scr. uptake: NR	NR	Counseling sessions were offered to provide education to prevent infection transmission, and HCV infected couples are encouraged to avoid marriage. No results on medical follow-up reported.
									Prevalence: 0.3% (250/74662; 95% CI: 0.3-0.4)**		
											Outcomes: RNA rate: NR Start treatment: NR SVR: NR
Dubois, F, 1994 [94]	NR	Healthy subject of routine medical check up	France (1.1%): Western part	Routine medical check up	5 weeks	ALT, HAV, HBV, HDV	Yes: elevated ALT levels (vs control group without elevated ALT)	NR	Scr. uptake: NR Prevalence: 4.9% (15/308; 95% CI: 3.0-7.9)*** Control group: 0.3% (1/308)	Most common risk factors: - Blood transfusion - History of IDU	Patients were referred to their family physician (no results reported). Outcomes:RNA rate: 73.3% (11/15) RNA rate control gr: 0% (0/1) Start treatment: NR SVR: NR
Roberts, J, 2010 [95] (abstract)	2009	MSM attending service and who were tested for HIV	UK (1.1%): Brighton	Local outreach services for HIV point of care testing	4 months	HAV, HBV, syphilis, HIV	No	NR	Scr. uptake: 66.2% (55/82)	NR	Subsequent attendance at STI services remained low. Follow-up of the HCV-infected person was not reported in detail. Outcomes: RNA rate: - Start treatment: NR SVR: NR
									Prevalence: 1.8% CHCV (1/55; 95% CI: 0.1-9.6)****		
Cohen, DE, 2006 [96]	2001	MSM	USA (1.9%): Greater Boston area	Community care facility	8 months	None	No	Yes	Scr. uptake: NR Prevalence: 11.5% (25/218; 95% CI: 7.9-16.4)***	Univariate regr. analysis: - HIV infection - HBV infection - Less receptive anal sex - Lifetime history of gonorrhoea - History of IDU - Reporting blood on shared cocaine straws - Crack cocaine use in prior 6 months	Patients were referred to their primary care provider (no results reported). Outcomes: RNA rate: NR Start treatment: NR SVR: NR
Campello, C, 2002 [97]	1994-1995	Individuals ages 17–67 years, currently employed in the processing and/or trade of food and beverages	Italy (1.1%): Lombardia region, administrative boundary of the former USL 22 (local health unit)	Periodic compulsory health check for the surveillance and control of diseases transmitted by the fecal-oral route as well as tuberculosis	14 months	None	No	NR	Scr. uptake: 77.6% (2154/2776) Prevalence: 3.3% (71/2154; 95% CI 2.6-4.1)***	Multivariable regr. analysis: - Age >50 years - Blood transfusion - History of IDU - Tattooing - <8 years of education - Female sex	Patients offered the possibility of undergoing a follow-up for the clinical and laboratory evaluation of hepatic involvement (no results reported). Outcomes: RNA rate: 71.8% (51/71) Start treatment: NR SVR: NR
Tafuri, S, 2010 [98]	2008	Asylum seekers without signs or symptoms in recent or remote past	Italy (1.1%): Bari	Asylum seeker center	3 months	HBV, HIV, syphilis	No	NR	Scr. uptake: 71.1% (529/744)	NR	All patients who tested positive were treated (no results were reported).
									Prevalence: 4.5% (24/529 ; 95% CI: 3.06-6.07)*		Outcomes: RNA rate: NR Start treatment: NR SVR: NR

Note: CI = confidence interval; NR = not reported; IDU = injecting drug use; HCV = hepatitis C virus; CHCV = chronic hepatitis C virus; HBV = hepatitis B virus; HAV = hepatitis A virus; HDV = hepatitis delta virus; HIV = human immunodeficiency virus; ALT = alanine aminotransferase; MSM = men who have sex with men; SVR = sustained virological response; PCR = polymerase chain reaction.

*HCV-antibody prevalence is considered suboptimal (data were collected before 1994 when sensitivity/specificity of tests was not optimal, or reactive HCV-antibody test results were not confirmed by immunoblot).

**The reliability of the reported HCV-antibody prevalence is undecided (data were collected after 1993, but the diagnostic tests are unspecified, or other than described below, or dried blood spots or oral fluid samples were used).

***HCV-antibody prevalence is considered valid; data were collected after 1993, and reactive HCV-antibody test results were confirmed by second or higher generation immunoblot assays from Ortho, Chiron, Novartis (RIBA), Innogenetics (LiaTek), Pasteur (DECISCAN HCV), Genelabs Diagnostics (HCV BLOT), or Mikrogen (recomBlot HCV IgG 2.0).

****HCV-antibody prevalence is considered valid, but reflecting chronic HCV infection (data were collected after 1993, and reactive HCV antibody test results were confirmed by PCR).

### Clinics for sexually transmitted diseases (n = 11)

#### Program characteristics

The majority (7/11) of the HCV screening programs in STD clinics were carried out in the USA (see Table 2). None of the studies reported the use of personal invitations or media to promote HCV screening. In five of the 11 programs, screening was limited to high-risk groups for HCV, varying from single groups (e.g., people with a history of IDU [64], or MSM [60]) to individuals from multiple risk groups, such as those who have had body piercing or tattooing in unsanitary conditions, transfusion recipients before 1987, or those who have had a needlestick injury [59]. None of the studies reported whether or not individuals were charged for screening, or whether anonymous participation in the screening program was possible. None of the programs used a comparison group for evaluation purposes. All programs used venipuncture for specimen collection.

#### Program outcomes

The number of individuals screened for HCV antibodies per program ranged from 618 to 90,424. Six of the 11 programs reported the screening uptake, which varied from 14.0% to 95.8%. The HCV prevalence varied from 0.1% to 28.0%. Of the five studies that limited HCV screening to HCV risk groups, four reported a high prevalence (>15%). In contrast, the prevalences in the six studies without a risk selection varied from 0.1% to 4.9%. In all programs at the STD clinics, a history of IDU was found in the risk profile of the identified HCV-infected individuals, or was found associated with HCV infection. None of the studies reported the proportion of HCV-infected patients that started treatment and/or reached SVR.

### General practitioners’ clinics (n = 12)

#### Program characteristics

The majority (8/12) of the HCV-screening programs in GP clinics were carried out in France (see Table 3). In most programs (9/12), screening was limited to HCV risk groups within the GP-patient population (specific migrant groups [46,47]; risk groups such as those with a history of IDU and recipients of blood transfusions before 1991 [70,72,73,75-78]). One program was carried out in a health care center that attracted people with poor access to health care, mostly migrants [44], and one was carried out in an area of low socioeconomic status [71]. In two of the 12 programs, individuals who were in the GP’s waiting room were approached and invited for screening [44,46], and five programs used media activities to attract individuals for screening. Of the 12 studies, four reported that screening was free of cost [70,73,78,99], whereas the others did not report participants’ costs for screening. Only the screening program among people with poor access to health care offered the possibility of anonymous screening [44].

In reference to screening procedures, all but two studies used venipuncture for specimen collection. Two screening programs used oral fluid HCV antibody tests, followed by blood tests for those who tested positive. The majority (9/12) of the programs focused solely on HCV screening. The three programs that screened predominantly migrants also included HBV screening [44,46,47].

#### Program outcomes

The number of individuals screened for HCV antibodies per program ranged from 117 to 15,952. Six of the 12 programs reported the screening uptake, which varied from 27.8% to 82.5%. The HCV prevalence varied from 0% to 30.8%. Of the 12 programs, three primarily screened migrants (prevalences 0%, 1.2% and 5.8%), seven used risk factors other than being a migrant as criteria for screening (prevalences 1.4% to 30.8%), and one was performed in an area of low socioeconomic status (prevalence 12.8%). In contrast, one program that did not use risk factors as screening criteria, and was not performed in an area of low socioeconomic status, found a relatively low prevalence of 0.4% [74]. We did not notice clear differences in screening uptake or HCV prevalence related to the use of media to attract individuals for screening. Further, we could not assess whether personally inviting individuals for screening or screening for more than just HCV could have influenced the screening uptake, since these studies did not report the screening uptake.

Four studies checked the results of their screening program against data collected in the same period in comparison clinics or data collected prior to the screening program. A study from the Netherlands concluded that the addition of primary care practice support leads to considerable improvements in medical awareness regarding HCV infection in primary care, which is likely to have a positive effect on case finding (that effect, however, could not be indisputably demonstrated) [76]. A study from France concluded that information and training that is adapted to GPs’ medical practice can lead to more active involvement of GPs in screening for HCV infection [77]. During the intervention the number of GPs that prescribed HCV screening increased, and more HCV-infected patients were detected compared with the year before. Another study from France compared two interventions in the GP clinic; GPs in intervention 1 prescribed HCV screening if HCV risk factors were identified during questioning of patients, whereas GPs in intervention 2 placed posters and leaflets on HCV risk factors in their waiting rooms to motivate patients at risk to discuss screening [78]. The numbers of tests prescribed by GPs was relatively low in both interventions, and outcomes of the two interventions with regard to the number of tests and the HCV prevalence were comparable. In a study from Scotland, HCV screening was offered to all GP visitors aged 30–54 years and 117 individuals were screened (prevalence: 12.8%, 15/117), whereas in a comparison clinic, where no intervention for HCV screening was introduced, no individuals were screened for HCV [71]. Two of these programs reported the proportion who started treatment (18% and 38%), but only one of these two programs reported the proportion of treated individuals (n = 2) who reached SVR (50%, n = 1) [71].

### Veterans affairs clinics (n = 5)

#### Program characteristics

Table 4 presents the five HCV screening programs in VA clinics in the USA. No personal invitations or media activities were reported. All screening programs limited the screening to risk groups within the veteran population. In one program, screening was limited to veterans who were admitted for an alcohol and noninjecting drug rehabilitation program [82], while other programs used an extensive list of risk factors, including history of drug use, blood transfusion prior to 1992, and Vietnam veteran. None of the programs reported whether or not individuals could participate anonymously, participants’ costs for screening, or a comparison group for evaluation purposes. All programs used venipuncture for specimen collection and screened solely for HCV.

#### Program outcomes

The number of individuals screened for HCV antibodies per program ranged from 338 to 12,485. The screening uptake was described in three of the five programs, varying from 41.9% to 99.4%. The HCV prevalence in most programs was around 5%, but the program among veterans who were admitted to an alcohol and noninjecting drug rehabilitation program was substantially higher (23.1%). The proportion of patients that started treatment was described in three studies and varied from 15% to 38%. Four studies reported the SVR rate among those who started treatment, ranging from 33% to 47%.

### Antenatal/obstetric/fertility clinics (n = 5)

#### Program characteristics

Of the five programs, three were carried out in the UK, one in the USA and one in Brazil (see Table 5). The programs targeted pregnant women, except for a British study in a fertility clinic that was targeted at couples. Media activities to promote the screening programs were described in only one of the five studies; this study used information leaflets to inform women about the screening program, and a personal invitation for participation by the midwife [87]. None of the programs used a risk assessment strategy to limit screening to those at risk. The programs did not report the possibility to screen anonymously, or a comparison group for evaluation purposes. The one program reporting screening costs was free of cost to participants [88].

With respect to screening procedures, all but one study used venipuncture for specimen collection. One study used DBS for anti-HCV screening and a second generation ELISA followed by HCV RNA testing using venous blood for confirmation [88]. In all but one program, participants were also screened for other infections (mainly HIV and HBV) or elevated liver enzymes (ALT/AST).

#### Program outcomes

The number of individuals screened for HCV antibodies per program ranged from 1,658 to 31,081. In the two studies reporting screening uptake, rates were very high (≥98%). In all but one program, the HCV prevalences were low, varying from 0.2% to 0.8%. In women at risk for perinatal complications, HCV prevalence was 4.6% [86]. In one of the five programs, results of the clinical follow-up and treatment were reported, showing that 67.9% of those identified with chronic HCV started treatment after delivery, and 80% of those who completed treatment achieved SVR [84].

### Psychiatric clinics (n = 3)

#### Program characteristics

Of the three HCV screening programs in psychiatric clinics, two were carried out in Australia, and one in the USA (see Table 6). One program aimed to evaluate whether screening by risk factors would be effective, and limited the HCV screening program in one unit to those with a history of IDU and those exposed to contaminated blood products, whereas in the other unit all patients were screened [90]. In the other two programs, no risk selection was used for participation in the screening program. One program promoted screening by using media [91]. The programs did not offer the possibility to screen anonymously, and did not report about participants’ costs for screening. Concerning screening procedures, all studies used venipuncture for specimen collection, and all studies exclusively screened for HCV.

#### Program outcomes

The number of individuals screened for HCV antibodies per program ranged from 36 to 98. All programs reported the screening uptake, varying from 20.5% to 100%. The HCV prevalences varied from 3.2% in the unit without pre-screening risk selection [90], to 41.7% in the unit where screening was limited to those who reported a history of IDU or exposure to contaminated blood products. Noninjecting drug use and history of IDU were reported as the main risk factors among the identified cases. All three programs referred the HCV-infected individuals to a specialist. Of interest, in two programs [90,91], post-test counseling addressing various topics (e.g., education about the illness, risk behavior, safe injection practices, secondary prevention) was also offered to those who reported risk factors but tested HCV negative.

One of the three studies reported that their program was based on psychosocial theory or knowledge about determinants facilitating participation in HCV screening programs. This program promoted screening by using leaflets outlining HCV, its risk factors, and the importance of screening, and used individually tailored pre- and post-test counseling that was adapted to individual knowledge and cultural understandings where appropriate [91]. Although the uptake of screening in that study was relatively low (20.5%), the prevalence was relatively high (19.7%), especially considering the fact that no prescreening risk selection was used.

### Other clinics (n = 7)

#### Program characteristics

In total, seven HCV screening programs were integrated in other clinics or services (see Table 7). These programs varied widely, from screening patients at an emergency health unit in France [92] to screening couples that wish to get married in Saudi Arabia [93]. Two programs targeted MSM; one in an outreach service for HIV point-of-care testing in the UK [95], and another in a community care facility in the USA [96].

The study in the USA reported the use of media activities to attract participants, wherein MSM were recruited through advertisements as well as by referral of medical staff [96]. Two of the seven studies used a prescreening selection; a program at an emergency health unit only screened those with a reported risk factor [92], and in France, only those with elevated ALT levels as determined during routine medical check-up were screened for HCV [94]. None of the programs reported about the possibility of anonymous screening, or a comparison group for evaluation purposes. The one study reporting about participants’ costs for screening mentioned that it was free [96]. With respect to screening procedures, all studies used venipuncture for specimen collection. Four of the seven programs also screened for other diseases (mainly HBV).

#### Program outcomes

The number of individuals screened for HCV antibodies per program ranged from 55 to 74,662. The screening uptake was reported in three out of seven programs, varying from 66.2% to 77.6%. The HCV prevalence varied from 0.3% in a mandatory premarital screening program in Saudi Arabia [93] to 11.5% in a program for MSM [96]. Only one study reported the number of patients that started treatment, which was zero [92].

## Discussion

This systematic review describes characteristics and outcomes of HCV screening programs in the general population, and includes 67 programs. In total, 24 of them were exclusively set up for the purpose of HCV screening, whereas 41 were integrated in already existing health care facilities (not aimed at HCV risk groups), and two programs used both an integrated and nonintegrated approach. Altogether, the programs that were published identified approximately 25,700 HCV-infected individuals. Clearly, large-scale, and long-term screening and referral programs are needed to address the HCV-related burden of disease in an era of potent therapy for HCV.

The programs were highly heterogenic in their organization, recruitment, and screening procedure, and the vast majority did not use a comparison group to assess the effectiveness of their screening program. Hence, we cannot draw firm conclusions as to which screening program strategy, or which program characteristic (e.g., free-of-cost vs. low-cost screening, anonymous vs. nonanonymous screening, use of particular media to promote screening) is more effective than another in attracting or motivating individuals for screening or in attracting those at higher risk for HCV. Screening programs that compare different recruitment and screening strategies are needed to gain insight into effectiveness of strategies and program characteristics.

In addition, many studies did not report program characteristics (e.g., the laboratory tests that were used). The same was true for screening uptake and follow-up data regarding HCV-related care, and even if reported, there was not much consistency (e.g., some reported the SVR rate among those who completed treatment, whereas others reported that treatment was ‘rather successful’). The underreporting and the lack of uniformity of data reporting greatly hinder the comparison of screening programs. Data reporting standards (see Parameters of screening programs) are needed to be able to compare screening program characteristics and outcomes in order to find out which factors are effective. If demonstration projects for HCV screening are not able to demonstrate direct effects in terms of number of individuals who tested positive for HCV that are linked to care, secondary evaluation measures could be used. For instance by monitoring rates of HCV screening and positivity in the hospital or referral laboratory and by comparing these data with data from previous years or from hospitals or laboratories that were not involved in the demonstration project.

In general, we noticed relatively high HCV prevalences in programs that used a prescreening selection based on HCV risk factors (especially in programs that used elevated ALT or a history of IDU as indications for HCV screening) or migrant status, in programs that were carried out in intermediate to high HCV-prevalence countries or regions, and in programs in psychiatric clinics. Also, relatively high HCV prevalences were found in nonintegrated programs in low HCV-prevalence countries that targeted the general population (see Additional file 1: Table S1, row 1–7), even without a prescreening risk assessment. These programs might have screened a self-referred population, and may have attracted those at risk of HCV in the general population, and therefore observed prevalences are higher than those in the general population. For the study by Hayashi etal (see Additional file 1: Table S1), screening was performed in a specific region in Japan with a presumably high HCV prevalence, explaining the very high prevalence that was found. In most studies, a history of IDU was the main risk factor among the identified HCV-infected individuals. In general, low HCV prevalences were found in programs that targeted health care workers, and in programs that were carried out in antenatal clinics. Programs in STD and GP clinics that did not use a prescreening risk selection also found relatively low HCV prevalences.

Only one study reported that the promotion of the screening program was based on theoretical insights or knowledge about determinants facilitating participation in screening programs. None of the studies reported the use of simple tools that may increase the screening uptake, such as reminder messages [100], or support with planning of when and how to get screened (i.e., creating implementation intentions [101]). In many studies, and especially those describing nonintegrated programs, the uptake of screening was not reported.

We found that integrated screening programs in general screened a larger number of individuals than did nonintegrated screening programs in low HCV-prevalence countries. Integrated screening programs have three advantages in that they do not have to attract their target population for screening, and they can use a facility that is familiar to the public. In addition, they can facilitate continuous screening and follow-up of individuals at relatively low cost, whereas nonintegrated programs offer screening usually for a limited period. On the other hand, integrated screening programs only reach those who have a reason to visit such facilities (unless media campaigns have been used to attract more people), whereas nonintegrated programs may attract a different risk population that otherwise would not be screened and do not perceive themselves at risk for HCV (i.e., the hidden population). We believe that both approaches are useful and complementary. In addition, since nonintegrated screening in general is more complex to organize, it may be efficient to screen for other diseases simultaneously, when risk groups overlap.

We identified several studies that did not confirm HCV antibody test results. Many of the identified programs targeted asymptomatic individuals in the general population with a relatively low HCV prevalence. In such populations, unconfirmed HCV antibody test results may include 35% (range: 15%–60%) false-positive test results [102]. Hence, the program outcomes that are reported may include a substantial degree of uncertainty, and should be interpreted with care. We like to emphasize that HCV screening programs should use validated screening methods, and describe the tests that were used when publishing the results of their screening programs.

As for study limitations, the majority of the screening programs that were evaluated in this review occurred in just a few countries (USA, UK, and France), most likely since this review was limited to studies published in English, French, German, Spanish, and Dutch. Therefore, the results may not be generalizable to other (non-Western) countries or countries with a higher HCV prevalence. With respect to publication bias, this review only includes screening programs that were published and programs that were successful in identifying HCV-infected individuals may have been more likely to be published. However, we did identify several programs in which no individuals were diagnosed. Furthermore, as identification of HCV-infected individuals serves a clinical goal and not necessarily a scientific goal, not all screening efforts have been evaluated or published. Our search identified several announcements of HCV screening activities [103,104] or cost-effectiveness evaluations of screening activities [105] that did not provide any further information about the screening program and/or outcomes. Further, our review describes several screening programs, but it cannot determine the efficiency and effectiveness of these screening programs in preventing future HCV-related morbidity and mortality. Measuring these effects of HCV screening programs is a challenge because randomized, controlled trials or comparison groups and decades of follow-up time are required. As an alternative method, mathematical modelling studies can be useful to estimate long-term effects of screening programs (e.g., [106,107]), especially when certain program outcomes (e.g., participation rate, prevalence) are used as parameters in the model. The efficiency and effectiveness of screening also depends on the uptake and outcomes of therapy and other preventive measures that may follow from diagnosis. Efficiency relates to the number needed to be screened to identify a treatable case of HCV. Surprisingly, most studies did not report such data, and merely mentioned that HCV-infected individuals were notified of their test result and referred for clinical care. Following Wilson and Jungners third screening principle [108], facilities for diagnosis and treatment should be available. This means that the screening program itself is as important as the efforts that are undertaken to bring identified patients into care and have them benefit from preventive measures and/or treatment. Hence, evaluation reports of screening programs should include clinical follow-up and systematically report outcomes.

A recent systematic review by Jones etal on the effectiveness and cost-effectiveness of interventions aimed at raising awareness of and/or increasing engagement in case finding and screening with high-risk groups for HCV and HBV and practitioners included only programs with a comparison group (e.g., randomized controlled trials, pre- and postintervention data, repeated cross-sectional studies) [109]. About half of the studies (12/25) included in that review were aimed at high-risk groups for HCV that are relatively easy to target, such as current IDU and incarcerated individuals, whereas our review includes studies that aimed to identify the hidden population of HCV-infected individuals. Jones etal identified drug services and primary care as settings in which interventions could effectively increase screening uptake. They also found that DBS screening in addition to venipuncture might increase HCV screening uptake in drugs services or prisons. In our review, a few studies reported the use of home collection tests, DBS, or oral fluid tests, but these studies did not demonstrate high screening uptake. Further insight into the effect of alternative noninvasive screening procedures on screening uptake is needed. As in our review, Jones etal concluded that improvement of health outcomes following diagnosis for those identified with chronic HCV deserves careful attention.

In the United States it has recently been recommended that all people born between 1945 and 1965 should be offered a one-time screening without prior risk assessment [110], since HCV prevalence in this group is four times greater than adults aged 20 years or older who were born outside of the birth cohort, and identifying this group through a high risk profile has been unsuccessful so far. More importantly, this screening strategy is estimated to be cost-effective assuming an uptake of 15% [106]. Although a relatively low uptake of 15% was assumed in the cost-effectiveness model in the USA, those who participate may not be representative for the total population of baby boomers. It may be that those at lower risk for HCV (i.e., the worried-well) could be more likely to respond. Thus, although it may be promising, the effectiveness of such an intervention needs to be demonstrated in practice. Nevertheless, it should be examined whether it is feasible and cost-effective to implement such a birth cohort screening in countries with a lower HCV prevalence.

## Conclusions

HCV infection has serious health implications and, at the start of the era of potent therapy for HCV, screening programs are not yet reaching all potentially infected individuals worldwide. Therefore more effective programs are urgently needed. This review identified 67 screening programs that targeted HCV risk groups that are hidden in the general population. Relatively high HCV prevalences were found in programs that used a prescreening selection based on a HCV risk profile or migrant status, in programs that were carried out in intermediate to high HCV-prevalence countries or regions, and in programs in psychiatric clinics. In general, low HCV prevalences were found in programs that targeted health care workers and pregnant women. The reported use of motivational communication based on theory and/or determinants facilitating screening, and tools to increase HCV screening uptake were virtually absent. Comparison of the screening programs was strongly hindered by the lack of reported data on screening uptake, program characteristics, the type of diagnostic tests used, and clinical outcomes. In addition, only a few programs used a comparison group to evaluate program effectiveness.

We suggest that for low HCV-prevalence populations, the use of prescreening selection criteria should be considered to increase efficiency. In addition, to be able to assess screening program effectiveness, programs using a comparison group are needed. To improve comparability of screening programs and outcomes, it is necessary for all programs to systematically report program characteristics, screening uptake, the type of diagnostic tests that were used, as well as clinical outcomes.

## Competing interests

The authors declare that they have no competing interests.

## Authors’ contributions

FRZ and ATU designed and performed the literature review, collected data from individual studies, participated in data interpretation, and drafted the manuscript. CWH and CHSBB participated in the study selection process, UD contributed to the study design and drafting of the manuscript. MWL supervised the study, and led the writing. MP conceived and supervised the study, participated in the study selection process, and led the writing. All authors read and approved the final manuscript.

## Pre-publication history

The pre-publication history for this paper can be accessed here:

http://www.biomedcentral.com/1471-2458/14/66/prepub

## Supplementary Material

Additional file 1: Table S1 Nonintegrated screening programs in low HCV-prevalence countries (≤2%) [23,46-54,111-123]. Click here for file
