# Supplementary material for: Outcomes of hepatitis C screening programs targeted at risk groups hidden in the general population: a systematic review
Source: BMC Public Health. 2014 Jan 22;14:66. doi: 10.1186/1471-2458-14-66 (PMC4016146; doi:10.1186/1471-2458-14-66)
Supplement: Additional file 1: Table S1 — Nonintegrated screening programs in low HCV-prevalence countries (≤2%) [23,46-54,111-123]. [file 1471-2458-14-66-S1.doc]

| **Table S1. Nonintegrated screening programs in low HCV-prevalence countries (≤2%)** | | | | | | | | | | | |
| --- | --- | --- | --- | --- | --- | --- | --- | --- | --- | --- | --- |
| **Program characteristics** | | | | | | | | | **Program outcomes** | | |
| **First author, year of publication** | **Calendar year of data collection** | **Population** | **Country and HCV prevalence according to CDC (23)** | **Setting of screening** | **Duration of screening program** | **Other**  **tests** | **Prescreening selection** | **Media activities** | **Screening uptake and anti-HCV prevalence**  **(95% CI)** | **Risk profile of identified HCV cases / Risk factors associated with HCV** | **Follow-up of HCV-infected individuals** |
| Kaur, S, 1996 (112) | 1992 | General population | USA (1.9%):  throughout the country | Mainly urban hospital centres | 4 days | HBV, ALT | No | Yes | Scr. uptake: appr. 90%  Prevalence:  7.0% (604/8650; 95% CI: 6.5-7.5) * | Multivariable regr. analysis  - History of IDU  - Hemodialysis  - Sex with IDU  - Blood transfusion  - Male gender  - Non-white/non-Hispanic  - Not vaccinated for HBV | Patients were referred to their physicians for further follow-up. Of 604 anti-HCV positives, 380 (62.9%) had elevated ALT levels. No results were reported.  Outcomes:  RNA rate: NR  Start treatment: NR  SVR: NR |
| D'Souza, RFC, 2004 (52) | 2003 | General population | UK (1.1%):  London | Walk-in clinic at the minor injuries unit at hospital | 4 days | Liver function tests | No | Yes | Scr. uptake: NR  Prevalence:  10.5% (2/19;95% CI:2.9-31.4) ** | NR | Patients were informed in person of the test results. No further follow-up data reported.  Outcomes:  RNA rate: NR  Start treatment: NR  SVR: NR |
| Bellentani, S, 1994 (113) | 1994 | General population aged 12-65 yrs | Italy (1.1%):  Northern Italy, cities Campogalliano and Cormons | Community-based screening | 2 years | ALT, AST, gamma-glutamyltranspeptidase, mean cell volume, platelet, erythrocyte and leukocyte counts, HBV | No | Yes | Scr. uptake: 68.1% (6917/10150)  Prevalence:  2.9% (199/6917; 95% CI: 2.5-3.3) * | NR | Patients with HCV antibodies underwent additional procedures (e.g., ultrasonography of the liver, liver biopsy when indicated). At the time of the writing, 10% had undergone liver biopsy. No results were reported.  Outcomes:  RNA rate: NR  Start treatment: NR  SVR: NR |
| Trepka, M.J, 2007 (53) | 2001- 2003 | General population | USA (1.9%):  Miami | Hepatitis screening clinic | 2.5 year | HBV | Yes, if traditional  risk factors apply ^b^ | NR | Scr. uptake: NR  Prevalence:  NR (269/NR) *** | Most common risk factor (%)  - History of IDU (54.7) | Of the anti-HCV positive clients, 20.8% (56/269) were reached by phone, and 44 were interviewed. Of those, 31 (70.5%) had seen a physician, of which 27 completed their medical evaluation. Of these, 3 completed treatment, 7 were still receiving treatment, 8 had not yet begun treatment, 7 did not need treatment, and 2 were no treatment candidates.  Outcomes:  RNA rate: NR  Start treatment: 37.0% (10/27)  SVR: NR |
| Fagundes, GD, 2008 (114) | 2005 | General adult urban population | Brazil (1%):  Santa Catarina, Criciuma | Public health campaign event | 1 day | None | No | Yes | Scr. uptake: NR  Prevalence:  2.2% (10/457; 95% CI: 1.2-4.0) * | Univariable regr. analysis: - High number of sex partners | In HCV-RNA positive samples, genotyping was performed for therapeutic reasons. No data about therapy reported.  Outcomes:  RNA rate: 70.0% (7/10)  Start treatment: NR  SVR: NR |
| Jimenez, FP, 2000 (51) | 1997-1998 | General population 15-70 years | Cuba (1.9%): Havana | House visits of all patients registered at a GP clinic | 17 months | None | Yes, history of blood transfusion | No | Scr. uptake: 100% (35/35)  Prevalence:  8.6% (3/35; 95% CI: 3.0-22.4) * | NR | All patients were followed and treated at a gastroenterology clinic (results were not reported)  Outcomes:  RNA rate: NR  Start treatment: NR  SVR: NR |
| Hayashi, J, 1995 (111) | 1993 | General population | Japan (2%):  Kyushu Island, Fukuoka Prefecture, 'H Village' | Village screening program | NR | HBV | No | Yes | Scr. uptake: 48.1% (2046/4250)  Prevalence:  19.7% (403/2046; 95% CI: 18.0-21.5) * | Most common risk factor (%)  - History of blood transfusion (11.9) | NR – the authors write that it is necessary to work out a strategy for the care of the many HCV-infected individuals in this village.  Outcomes:  RNA rate: 82.9% (334/403)  Start treatment: NR  SVR: NR |
| Uddin, G, 2010 (46) ^a^ | NR | Immigrants from the Indian sub-continent (India, Bangladesh or Pakistan) | UK (1.1%):  East London, West London, Walsall, Sandwell, Bradford | Public meetings and testing sessions in community centers (and GP clinic, see Table 2b) | NR | Oral fluid HCV, HBV | No | Yes | Scr. uptake: NR  Prevalence:  1.6% (75/4,833; 95% CI:1.2-1.9) at community centers ** | Multivariable regr. analysis  - Pakistani  - Shorter length of stay in the UK  - Being tested in East London | Patients were offered an appointment with the local treating physician for confirmation blood testing; 57/75 attended.  Outcomes:  RNA rate: 96.5% (55/57)  Start treatment: NR  SVR: NR |
| Kallman, JB, 2010 (47) ^a^ | NR | Immigrants from Vietnam | USA (1.9%):  Northern Virginia | General health screening at Asian health fairs (and GP clinic, see Table 2b) | NR | HBV | No | NR | Scr. uptake: NR  Prevalence:  5.2% (4/77, 95% CI: 2.0-12.6) at health fairs ** | Univariable regr. analysis:  - Elevated AST | Patients were seen by their primary care givers for further management, or referred for further follow-up and treatment (no results were reported).  Outcomes:  RNA rate: NR  Start treatment: NR  SVR: NR |
| Hwang, JP, 2010 (50) | 2006 | Asian Americans (predominantly) | USA (1.9%):  Houston | Local community health fair | 1 day | HBV | No | Yes | Scr. uptake: >20% (202/>1000, of whom 118 Asian Americans)  Prevalence:  5.9% (7/118, 95% CI: 2.9-11.7) *  Vietnamese: 15.4% other Asian: 1.3% | NR | Of 7 patients, one was lost to follow-up, and 6 were tested for HCV RNA. Those who tested positive were referred to a hepatologist. Access to care was not confirmed, and follow-up data were not reported.  Outcomes:  RNA rate: 83.3% (5/6)  Start treatment: NR  SVR: NR |
| Batash, S, 2008 (115) | NR | Immigrants in NYC from former Sovjet Union | USA (1.9%):  NYC | Community based screening | 3 days | None | No | Yes | Scr. uptake: NR  Prevalence:  28.3% (80/283, 95% CI:23.0-33.5) * | Only available for small subset of cases (97/283)  Multivariable regr. analysis:  - intramuscular injections  - blood transfusions | HCV RNA and ALT testing was only done in the 27 individuals that were identified at screening day 2 and 3.  Outcomes:  RNA rate: 66.7% (18/27)  Start treatment: NR  SVR: NR |
| ARèS 92, 2004 (116) | 2004 | Guest workers from Africa | France (1.1%):  Hauts-de-Seine | Health check in rental apartments for guest workers | 3 days | Clinical and dental examination, a chest X-ray and blood tests: fasting glucose, cholesterol, triglycerides, and serologies: HBV, syphilis and HIV | No | Yes | Scr. uptake: 35.6% (110/309)  Prevalence:  0.9% (1/110; 95% CI:0.04-5.0) ** | NR | For all patients specific management was started (results were not reported).  Outcomes:  RNA rate: NR  Start treatment: NR  SVR: NR |
| Goetz, AM, 1995 (117) | NR | Health care workers (physicians, dentists, nurses and laboratory personnel) with very high, high, and low risk for potential exposure to hepatitis C through the handling of blood and body fluids. | USA (1.9%):  Pittsburgh | Two hospitals that do liver transplantations: the Veterans Affairs Medical Center and the Presbyterian University Hospital | NR | None | No | NR | Scr. uptake: NR  Prevalence:  Overall: 1.2% (3/241; 95% CI: 0.4-3.6) CHCV. ****  In HCW involved with liver transplantations: 5.3% (3/57, 95%CI: 1.3-15.5%)  versus 0% in the HCW at lower risk. | Univariable regr. analysis:  - Working at liver transplant operating room | Clinical evaluation and counseling for those who were not anonymous (results were not reported).  Outcomes:  RNA rate: -  Start treatment: NR  SVR: NR |
| Thomas, DL, 1993 (118) | 1991 | Health care personnel | USA (1.9%):  East Baltimore | Hospital | 9 months | HBV | No | Yes | Scr. uptake: >90%  Prevalence:  0.7% (7/943; 95% CI: 0.4-1.5) * | Most common risk factor (%)  - Blood transfusion (14.3) | Patients were offered consultation (results were not reported).  Outcomes:  RNA rate: NR  Start treatment: NR  SVR: NR |
| Panlilio, AL, 1995 (119) | 1992 | Surgeons | USA (1.9%):  throughout two metropolitan areas | 21 hospitals in two metropolitan areas | 7 months | HBV, HIV | No | Yes | Scr. uptake: 26.7% (770/2887)  Prevalence:  0.9% (7/770; 95% CI: 0.4-1.9) * | None identified | Patients were offered post-test-counseling (results were not reported).  Outcomes:  RNA rate: NR  Start treatment: NR  SVR: NR |
| Upfal, MJ, 2001 (120) | NR | Firefighters, police and EMS | USA (1.9%):  Detroit | Survey among firefighters, police, emergency medical service (EMS) personnel | NR | None | No | Yes | Scr. uptake: 42.9% (2447/5700)  Prevalence:  1.1% (28/2447; 95% CI: 0.8-1.6) * | Multivariable regr. analysis:  - EMS personnel, fire fighters  - Guilty about drinking  - Surgery<1990  - Older age  - Life dissatisfaction | Patients were advised of the need and available resources for follow-up confirmatory testing, counseling, and preventive and medical care (results were not reported).  Outcomes:  RNA rate: NR  Start treatment: NR  SVR: NR |
| Datta, S, 2003 (49) | 1999 | Active and retired fire fighters from local union | USA (1.9%):  Philadelphia | Home testing screening project | NR | None | No | NR | Scr. uptake:48.3% (2127/4400)  Prevalence:  3.6% (77/2127, 95% CI: 2.9-4.5%) ** | Multivariable regr. analysis:  -Blood transfusion before 1992  - History of illegal drug use  - Black race | Patients received their results by phone.  Outcomes:  RNA rate: NR  Start treatment: NR  SVR: NR |
| Gershon, RRM, 1995 (121) | NR | Funeral service practitioners | USA (1.9%):  Maryland | Testing on appointment | NR | HIV, HBV | No | NR | Scr. uptake: 49.6% (130/262)  Prevalence:  0% (0/130; 95% CI:0-2.9) ** | Not applicable (none identified) | Not applicable (none identified) |
| Torda, AJ, 2008 (122) | 2002-2005 | First-year medical students | Australia (2%):  New South Wales | Mandatory vaccination and screening program in a vaccination clinic | 4 years | HIV, HBV, measles, mumps, rubella, and varicella-specific IgG antibodies | No | NR | Scr. uptake: 85.0% (733/862)  Prevalence:  0.5% (4/733; 95% CI:0.2-1.4) ** | NR | Patients were appropriately followed-up (results other than chronicity rate were not reported).  Outcomes:  RNA rate: 50.0% (2/4)  Start treatment: NR  SVR: NR |
| Plard, C, 2007 (54) | 2005 | Underprivileged people at risk of HIV: IDU, illegals, etc. | France (1.1%):  Paris | Outreach screening compared with records of individuals that came to a free and anonymous hospital-based HIV testing clinic | 1 year | HIV, HBV, syphilis | No | NR | Scr. uptake outreach: 98.6% (427/433)  Prevalence (outreach):  4.9% (21/427; 95% CI:3.2-7.4)  Prevalence (clinic): 1.6% (7/427; 95%CI:0.8-3.3) ** | NR | NR  Outcomes:  RNA rate: NR  Start treatment: NR  SVR: NR |
| Boyce, DEC, 2009 (48) | 2006 | Homeless individuals | USA (1.9%):  Hawaii, Oahu | Hepatitis health fair organized in a shelter for homeless people | 1 day | HBV | No | NR | Scr. uptake: unclear  Prevalence:  7.5% (3/40; 95% CI: 2.6-19.9) ** | Most common risk factor (%)  - Jail time (100)  - History of IDU (67)  - Tattoos (67)  - Piercings (67)  - Snorting drugs (33)  - Blood transfusion (33)  - Sex partner with HCV infection (33) | Participants were provided with information about available health care resources in the event that they tested positive (results were not reported).  Outcomes:  RNA rate: NR  Start treatment: NR  SVR: NR |
| Arumainayagam J, 2009 [123) | 2007 | Asymptomatic MSM (and symptomatic MSM who declined referral to the genitourinary medicine clinic) | UK (1.1%):  Walsall | Outreach sessions at the sauna | 1 year | HBV, HIV, syphilis, chlamydia, gonorrhea | No | NR | Scr. uptake: NR  Prevalence:  2.2% CHCV (2/91; 95% CI: 0.60-7.66) ** | NR | Patients were referred to and attended their local genitourinary medicine clinic (results were not reported).  Outcomes:  RNA rate: -  Start treatment: NR  SVR: NR |

Note: CI = confidence interval; NR = not reported; IDU = injecting drug use; HCV = hepatitis C virus; CHCV = chronic hepatitis C virus; HBV = hepatitis B virus; HIV = human immunodeficiency virus; ALT = alanine aminotransferase; AST = aspartate aminotransferase; MSM = men who have sex with men; HCW = health care worker; SVR = sustained virological response; PCR = polymerase chain reaction

* HCV-antibody prevalence is considered suboptimal (data were collected before 1994 when sensitivity/specificity of tests was not optimal, or reactive HCV-antibody test results were not confirmed by immunoblot).

** The reliability of the reported HCV-antibody prevalence is undecided (data were collected after 1993, but the diagnostic tests are unspecified, or other than described below, or dried blood spots or oral fluid samples were used).

*** HCV-antibody prevalence is considered valid; data were collected after 1993, and reactive HCV-antibody test results were confirmed by second or higher generation immunoblot assays from Ortho, Chiron, Novartis (RIBA), Innogenetics (LiaTek), Pasteur (DECISCAN HCV), Genelabs Diagnostics (HCV BLOT), or Mikrogen (recomBlot HCV IgG 2.0).

**** HCV-antibody prevalence is considered valid, but reflecting chronic HCV infection (data were collected after 1993, and reactive HCV antibody test results were confirmed by PCR).

^a^ These programs combined a nonintegrated screening approach with integrated screening at the GP clinic (see Table 3). Here only results of the nonintegrated screening are presented.

^b^ History of IDU, receiving blood transfusions or organ transplants prior to July 1992, clotting factor concentrates produced before 1987, being notified to have received HCV-positive blood, ever on chronic hemodialysis, persistently elevated ALT levels, ever exposed to HCV-positive blood through needlestick injuries, born to an HCV-positive woman.
